# U.S. POINTER Study: Adherence to a Multidomain Health Behavior Change Intervention

**DOI:** 10.1002/alz70860_098285

**Published:** 2025-12-23

**Authors:** Jennifer Ventrelle, Jeffrey A. Katula, Sarah Graef, Katelyn R Garcia, Allison Heinrich, Sharon Wilmoth, Mina Ghadimi Nouran, Katherine Lambert, Claire E Day, Olivia Matongo, Ann Marie McDonald, Richard Elbein, Susan Antkowiak, Kellie Holley, Rose Treviño‐Whitaker, Kristie Miller, Kathryn Demos, Justin Wade Johnson, John E Nichols, Kristin R Krueger, Christy C Tangney, Martha Clare Morris, Laura D Baker, U.S. POINTER Study Group

**Affiliations:** ^1^ Rush University Medical Center, Chicago, IL, USA; ^2^ Wake Forest University, Winston Salem, NC, USA; ^3^ Rush University, Chicago, IL, USA; ^4^ Wake Forest University School of Medicine, Winston‐Salem, NC, USA; ^5^ Wake Forest University, Winston‐Salem, NC, USA; ^6^ Wake Forest University School of Medicine, Winston Salem, NC, USA; ^7^ Alzheimer's Association, Western Carolina Chapter, Charlotte, NC, USA; ^8^ Alzheimer's Association, San Jose, CA, USA; ^9^ Alzheimer's Association, Illinois Chapter, Chicago, IL, USA; ^10^ Alzheimer's Association, Houston, TX, USA; ^11^ Alzheimer's Association, Watertown, MA, USA; ^12^ UC Davis Health, Sacramento, CA, USA; ^13^ Kelsey Research Foundation, Houston, TX, USA; ^14^ Brown University, Providence, RI, USA; ^15^ Wake Forest School of Medicine, Winston‐Salem, NC, USA; ^16^ Rush Institute for Healthy Aging, Chicago, IL, USA

## Abstract

**Background:**

U.S. POINTER was a multisite randomized controlled trial testing the impact of multidomain lifestyle intervention on cognitive function in cognitively unimpaired older adults at risk for cognitive decline and dementia. Here we present the intervention adherence data.

**Methods:**

U.S. POINTER randomly assigned over 2000 participants to one of two intervention arms, both targeting physical activity, nutrition, cognitive/social challenge and cardiometabolic risk reduction, but differing in intensity and accountability. The intervention prescription for the Structured (STR) arm included frequent peer team meetings (weekly for 16 weeks, bimonthly for 2 months, monthly thereafter)**,** a structured exercise program using community facilities, a nutrition prescription modeled on the MIND diet, BrainHQ computerized cognitive training, regular cognitive/social challenge, and frequent monitoring of cardiometabolic health. The Self‐guided (SG) arm included a total of six peer team meetings, general health education covering all intervention domains, and gift cards to encourage healthy behaviors. Treatment fidelity assessments, guided by recommendations of the NIH Behavior Change Consortium, were conducted via annual site visits. Implementation of the intervention protocol was regularly monitored via monthly meetings with site intervention leadership to ensure consistency across study sites.

**Results:**

Adherence tracking for the STR intervention arm included team meeting attendance (%), Fitbit activity (mean very active minutes/week), self‐reported exercise (aerobic, resistance, and balance mean minutes/week), MIND diet adherence (mean score/week), cognitive training (mean BrainHQ levels completed/week), cognitive/social activity (% of goal completed/week), and blood pressure checks (% of goal completed/month). SG intervention adherence was tracked using team meeting attendance. This topline presentation of U.S. POINTER's intervention adherence will provide metrics for the entire cohort and by subgroups based on age, biological sex, race, ethnicity, APOE genotype, baseline cardiometabolic health status, and baseline cognitive function.

**Conclusion:**

U.S. POINTER's adherence metrics obtained in an ethnoracially and geographically diverse cohort (31% from underrepresented groups at 5 sites across the U.S.) will significantly inform our understanding of how to effectively facilitate behavior change to support brain health for older Americans at risk for Alzheimer's disease and related dementia.

